# Supplementary material for: Reduced Rank Regression-Derived Dietary Patterns Related to the Fatty Liver Index and Associations with Type 2 Diabetes Mellitus among Ghanaian Populations under Transition: The RODAM Study
Source: Nutrients. 2021 Oct 20;13(11):3679. doi: 10.3390/nu13113679 (PMC8620643; doi:10.3390/nu13113679)
Supplement: Supplementary file 1 [file nutrients-13-03679-s001.zip › nutrients-1377630-supplementary.pdf]

Supplementary data

Table S1. Sensitivity analysis excluding study participants with self-reported diabetes. Percentage of explained variation in food intake and factor loadings of RRR-derived dietary pattern scores related with Fatty Liver Index among men and women.

**Food group**	**Men (n = 1366)**		**Women (n = 2321)**	
	**Explained variation (%)**	**Factor loading**	**Explained variation (%)**	**Factor loading**
Poultry	**29.63**	**0.32**	17.08	0.30
Coffee and tea	**20.66**	**0.27**	**20.64**	**0.33**
Cereal	19.81	0.26	14.60	0.28
Condiments	19.06	0.26	15.44	0.29
Potatoes	14.01	0.22	1.81	0.10
Margarine	7.28	0.16	11.92	0.25
Alcohol	9.61	0.18	6.12	0.18
Fermented maize products	17.98	-0.25	19.73	-0.32
Refined and cereal	**25.21**	**-0.30**	11.48	-0.25
Roots, tubers & plantain	**27.56**	**-0.31**	**21.68**	**-0.34**
Palm oil	**31.18**	**-0.33**	15.70	-0.29
**Total**	9.6		6.3	

Factor loadings are correlations between food groups and the dietary pattern score. Figures in bold represent food groups with relevant contributions to the dietary pattern score (≥0.20% explained variation in the factor loadings for either males or females).

Table S2. Associations of the FLI-related and RRR-derived dietary pattern scores with type 2 diabetes among males and females[1].

**Model**	**Odds Ratio (95% confidence interval)**						
	**Q1**	**Q2**	Q3	Q4	Q5	P for trend	per 1 score-SD
Men							
Diabetes/Control	17/256	22/251	31/243	46/227	43/230		
Crude	1 (reference)	1.30 (0.69,2.47)	2.28 (1.27,4.11)	3.25 (1.85,5.74)	3.57 (2.04,6.27)	<.0001	1.70 (1.43,2.02)
Model 1	1 (reference)	1.38 (0.71,2.69)	2.12 (1.10,4.08)	2.36 (1.13,4.96)	2.43 (1.14,5.16)	0.02	1.48 (1.16,1.88)
Model 2	1 (reference)	1.22 (0.62,2.41)	1.84 (0.95,3.57)	2.09 (0.99,4.43)	2.22 (1.02,4.86)	0.033	1.51 (1.16,1.98)
Women							
Diabetes/Control	25/439	38/426	32/433	47/417	47/417		
Crude	1 (reference)	1.84 (1.11,3.06)	1.52 (0.90,2.57)	2.36 (1.44,3.86)	2.66 (1.64,4.32)	<.0001	1.32 (1.15,1.52)
Model 1	1 (reference)	1.87 (1.11,3.14)	1.53 (0.89,2.64)	2.45 (1.45,4.15)	2.67 (1.51,4.70)	0.0006	1.31 (1.10,1.55)
Model 2	1 (reference)	1.54 (0.91,2.62)	1.24 (0.71,2.16)	1.99 (1.16,3.40)	2.31 (1.30,4.10)	0.003	1.27 (1.07,1.52)

[1]Odds ratios (ORs), 95% confidence intervals (CIs) were calculated by logistic regression. p-values for trend were calculated by modelling the median of the dietary pattern scores as the independent variable. Model 1: adjusted for age (years) and study site (categorical).Model 2: Model 1 + education (4 categories), energy intake (kcal/d), smoking (yes/no), physical activity (METs-h/week), alcohol (alcohol/day).Type 2 diabetes was deinfed as HbA1c ≥48mmol/mol or self-reported diabetes or use of glucose-lowering medication.

Table S3. Pearson correlations between the biomarker-related dietary pattern score, food intake frequencies, and NAFLD biomarkers (log-transformed) among men (n=1,366).

**Food groups**	**Cholesterol**		**LDL-Cho**		**HDL-Cho**		**AST**		**ALT**		**GGT**		**Triglycerides**		**CRP**	
	**r**	**partial r**	**r**	**partial r**	**r**	**partial r**	**r**	**partial r**	**r**	**partial r**	**r**	**partial r**	**r**	**partial r**	**r**	**partial r**
Whole grain cereals	0.10	-0.03	0.21	-0.03	0.13	0.02	-0.20	-0.01	-0.01	-0.03	-0.05	-0.05	-0.12	-0.08	0.00	0.05

Poultry	0.11	0.00	0.10	0.00	0.11	0.04	-0.14	0.02	0.05	0.02	-0.02	-0.01	-0.06	-0.03	-0.07	-0.04
Dairy products	0.10	0.05	0.09	0.04	0.13	0.08	-0.11	-0.01	0.02	0.00	-0.07	-0.06	-0.09	-0.05	-0.03	-0.01
Coffee & tea	0.08	-0.05	0.06	-0.05	0.11	0.00	-0.20	-0.02	-0.02	-0.02	-0.02	-0.01	-0.04	0.01	-0.05	-0.01
Condiments	0.11	0.04	0.10	0.03	0.11	0.04	-0.05	0.05	0.09	0.05	-0.04	-0.02	-0.05	-0.01	-0.05	-0.02
Potatoes	0.12	0.03	0.09	0.01	0.14	0.05	-0.10	0.06	0.05	0.06	0.03	0.04	-0.01	0.05	-0.05	-0.02
Margarine	0.04	-0.05	0.04	-0.04	0.05	-0.03	-0.16	-0.04	-0.01	-0.01	-0.02	-0.02	-0.05	-0.02	-0.06	-0.04
Olive oil	0.05	-0.02	0.06	-0.01	0.06	0.00	-0.11	-0.01	0.04	0.03	-0.06	-0.05	-0.07	-0.05	-0.05	-0.03
Palm oil	-0.13	-0.01	-0.11	0.00	-0.12	-0.04	0.15	-0.02	-0.04	-0.02	0.02	0.01	0.05	0.01	0.07	0.04
Roots, tubers & plantain	-0.12	-0.01	-0.12	-0.02	-0.09	-0.03	0.14	0.01	-0.02	0.02	0.08	0.08	0.07	0.05	0.00	-0.03
Fermented maize products	-0.10	0.01	-0.09	0.00	-0.07	0.03	0.18	0.03	-0.01	-0.01	0.02	0.01	0.03	-0.01	0.04	0.01
Vegetarian mixed dishes	-0.03	0.05	-0.01	0.06	-0.08	0.00	0.13	0.01	0.06	0.07	0.04	0.03	0.07	0.03	0.07	0.04
Fish	0.02	0.05	0.05	0.07	-0.08	-0.02	0.07	0.01	0.03	0.02	0.05	0.04	0.03	-0.01	0.03	0.02

Partial correlation coefficients are adjusted for age, body mass index, and study site. Only food groups with factor loadings ≥0.15 are shown..

Table S4. Pearson correlations between the biomarker-related dietary pattern score, food intake frequencies, and NAFLD biomarkers (log-transformed) among women (n = 2,321).

Food group	Cholesterol		LDL-Cho		HDL-Cho		AST		ALT		GGT		Triglycerides		CRP	
	r	partial r	r	partial r	r	partial r	r	partial r	r	partial r	r	partial r	r	partial r	r	partial r
Whole grain cereals	-0.01	-0.04	-0.02	-0.03	0.15	0.00	-0.17	0.06	-0.02	0.07	-0.01	0.01	-0.21	-0.09	0.07	-0.02
Poultry	-0.03	-0.04	-0.05	-0.04	0.15	0.02	-0.24	-0.04	-0.08	-0.01	-0.04	-0.04	-0.19	-0.04	-0.03	0.00
Dairy products	0.00	0.01	-0.01	0.01	0.12	0.04	-0.11	0.01	-0.03	0.02	-0.01	0.01	-0.15	-0.06	-0.06	-0.04
Coffee & tea	0.01	-0.01	-0.02	-0.03	0.21	0.05	-0.27	-0.04	-0.13	-0.05	-0.03	-0.03	-0.19	-0.03	-0.03	0.01
Condiments	-0.02	0.01	-0.03	0.01	0.15	0.02	-0.22	0.00	-0.09	-0.01	-0.01	0.02	-0.20	-0.03	0.00	0.03
Potatoes	-0.06	-0.06	-0.08	-0.07	0.16	0.00	-0.18	0.01	-0.06	0.02	-0.03	-0.01	-0.19	-0.05	-0.02	0.03
Margarine	-0.01	-0.03	-0.02	-0.03	0.10	-0.01	-0.14	0.01	-0.05	0.01	-0.04	-0.04	-0.14	-0.05	-0.05	-0.03
Olive oil	-0.01	-0.03	-0.03	-0.04	0.15	0.08	-0.11	0.00	-0.05	-0.01	-0.04	-0.04	-0.17	-0.11	-0.06	-0.04
Palm oil	-0.03	-0.02	-0.02	-0.03	-0.17	-0.03	0.20	0.01	0.08	0.02	0.02	0.02	0.18	0.05	0.03	0.01
Roots, tubers & plantain	-0.06	-0.07	-0.04	-0.06	-0.14	-0.05	0.15	-0.02	0.05	-0.01	0.02	0.02	0.10	-0.02	0.00	-0.01
Fermented maize products	-0.04	-0.01	-0.03	-0.02	-0.09	0.02	0.11	-0.03	0.03	-0.03	-0.02	-0.01	0.07	-0.01	0.03	0.02
Vegetarian mixed dishes	0.08	0.09	0.09	0.08	-0.11	0.00	0.20	0.06	0.08	0.03	0.05	0.05	0.18	0.07	0.03	0.00
Fish	0.10	0.10	0.13	0.12	-0.10	-0.05	0.09	0.04	0.03	-0.01	0.06	0.05	0.12	0.07	0.06	0.03

Partial correlation coefficients are adjusted for age, body mass index, and study site. Only food groups with factor loadings ≥0.15 are shown.

Table S5. Sensitivity analysis excluding study participants with self-reported diabetes. Percentage of explained variation in food intake and factor loadings of RRR-derived dietary pattern scores, and explained variation in NAFLD biomarkers and response weights among men and women.

Food group	Men (n =1,250)		Women (n = 2,178)	
	Explained variation (%)	Factor loading	Explained variation (%)	Factor loading
Whole grain cereal	34.3	0.32	24.93	-0.26
Coffee tea	25.07	0.28	44.64	-0.35
Condiments	21.03	0.25	33.11	-0.3
Dairy products	23.24	0.26	14.13	-0.2
Fermented maize products	22.09	-0.26	5.58	0.12
Margarine	16.35	0.22	15.2	-0.21
Olive oil	14.54	0.21	19.13	-0.23
Palm oil	30.83	-0.31	26.89	0.27
Potatoes	18.69	0.24	27.82	-0.28
Poultry	24.99	0.27	34.85	-0.31
Roots, tubers & plantain	30.95	-0.31	12.26	0.18

Vegetarian mixed dishes	9.38	-0.17	27.32	0.28
**Total**	**11.03**		**12.02**	
**Biomarker**	**Explained variation (%)**	**Response weight**	**Explained variation (%)**	**Response weight**
Cholesterol	5.46	0.46	0.25	0.08
LDL-Cholesterol	5.47	0.46	0.86	0.15
HDL-Cholesterol	2.84	0.33	8.31	-0.48
AST	7.76	-0.55	12.6	0.59
GGT	2.67	-0.32	0.39	0.10
Triglycerides	1.5	-0.24	10.93	0.55
C-reactive protein	0.3	-0.11	0.37	0.10
ALT	0.07	0.05	2.35	0.26
**Total**	**3.25**		**4.51**	

Table S6. Associations of the biomarker-related and RRR-derived dietary pattern scores with type 2 diabetes among males and females[1.]

	Odds ratio (95% confidence interval)						
	**Q1**	**Q2**	Q3	Q4	Q5	P for trend	per 1 score-SD
Men							
Diabetes/Control	15/258	27/246	33/241	47/226	37/236		
Crude	1 (reference)	1.72 (0.92,3.2)	2.65 (1.47,4.79)	3.80 (2.14,6.74)	2.89 (1.61,5.21)	<.0001	1.46 (1.24,1.72)
Model 1	1 (reference)	1.71 (0.89,3.29)	2.22 (1.13,4.36)	2.34 (1.08,5.07)	1.59 (0.73,3.46)	0.61	1.12 (0.88,1.43)
Model 2	1 (reference)	1.62 (0.84,3.14)	1.98 (0.99,3.96)	2.00 (0.91,4.40)	1.38 (0.61,3.09)	1.23	1.09 (0.84,1.41)
Model 3	1 (reference)	1.44(0.74,2.79)	1.53 (0.75,3.10)	1.52 (0.68,3.41)	1.07 (0.47,2.44)	1.17	1.01 (0.78,1.32)
Women							
Diabetes/Control	37/427	38/426	36/429	36/430	44/420		
Crude	1 (reference)	0.98 (0.64,1.50)	0.79 (0.50,1.24)	0.91 (0.59,1.40)	1.02 (0.67,1.57)	0.96	1.02 (0.89,1.17)
Model 1	1 (reference)	1.19 (0.76,1.87)	1.36 (0.74,2.52)	1.52 (0.80,2.87)	1.51 (0.79,2.88)	0.23	1.30 (1.04,1.63)
Model 2	1 (reference)	1.12 (0.70,1.78)	1.17 (0.61,2.24)	1.25 (0.63,2.47)	1.25 (0.63,2.48)	0.55	1.24 (0.97,1.58)
Model 3	1 (reference)	1.08 (0.67,1.73)	1.14 (0.59,2.20)	1.27 (0.64,2.55)	1.31 (0.65,2.63)	0.40	1.27 (0.99,1.62)

[1]Odds ratios (ORs), 95% confidence intervals (CIs) were calculated by logistic regression. p-values for trend were calculated by modelling the median of the dietary pattern scores as the independent variable. Model 1: adjusted for age (years) and study site (categorical). Model 2: Model 1 + education (4 categories), energy intake (kcal/d), smoking (yes/no), physical activity (METs-h/week), alcohol (alcohol/day). Model 3: Model 2 + BMI (kg/m^2), waist circumference (cm).Type 2 diabetes was deinfed as HbA1c ≥48mmol/mol or self-reported diabetes or use of glucose-lowering medication.

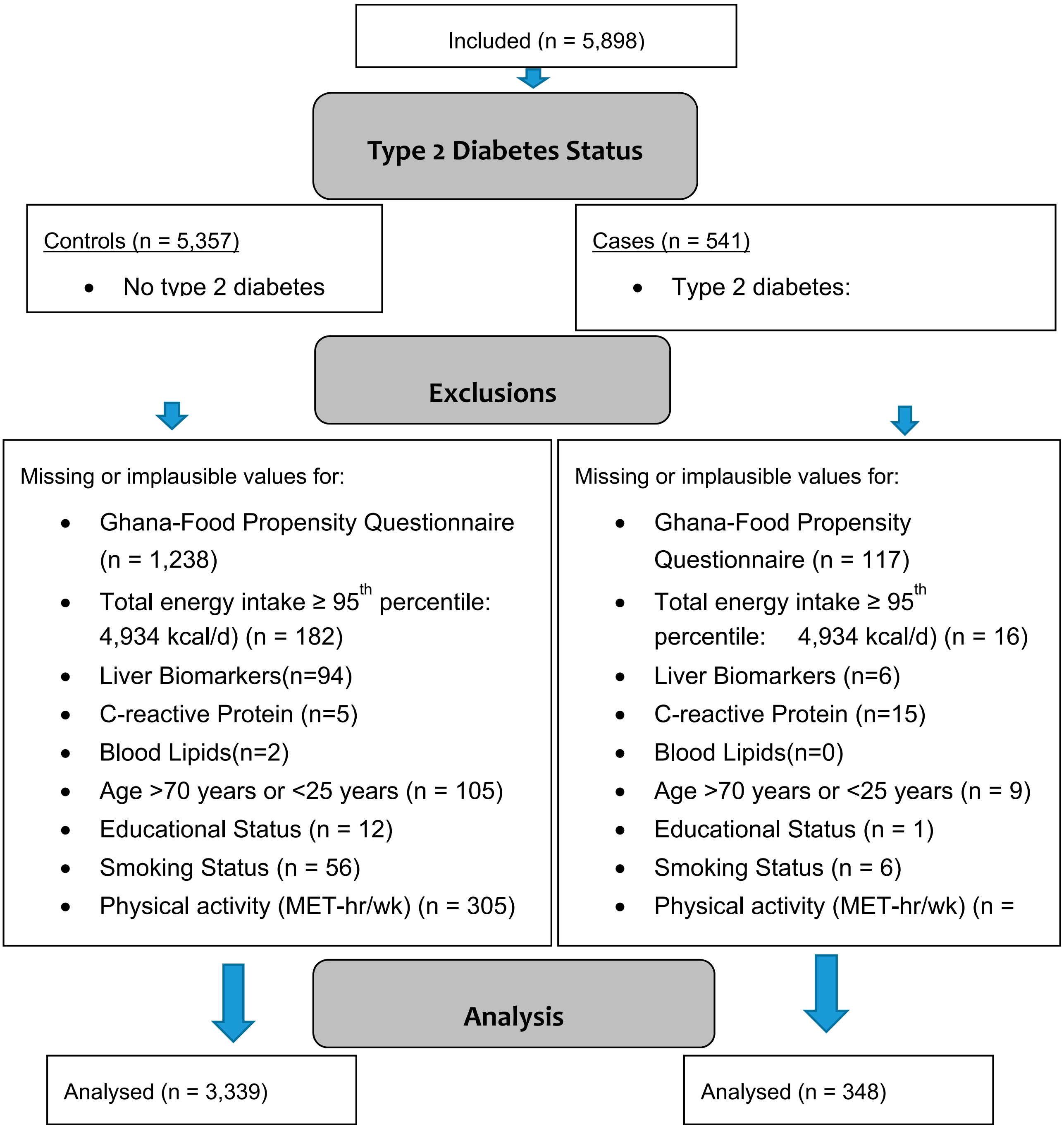

Figure S1. present the flow diagram of excluded participants because of missing or implausible data resulting in a simple size of 3687 participants for the characterization of dietary pattern.
